# Procaine Regulates the STAT3/CCL5 Axis and Inhibits Microglia M1 Polarization to Alleviate Complete Freund’s Adjuvant Rats Pain Behavior

**DOI:** 10.1523/ENEURO.0303-24.2024

**Published:** 2024-12-06

**Authors:** Yu Sun, Kai Zhang, Chen Li, QingDong Wang, Rongjia Zang

**Affiliations:** ^1^Department of Anesthesiology, The First Affiliated Hospital of Jiamusi University, Jiamusi, Heilongjiang Province 154002, P.R. China; ^2^Tuberculosis Department one ward, PLA General Hospital Eighth Medical Center, Beijing 100091, P.R. China; ^3^Jiamusi University, Jiamusi, Heilongjiang Province 154002, P.R. China

**Keywords:** microglia M1 polarization, neuropathic pain, procaine, STAT3/CCL5

## Abstract

Neuropathic pain (NP) caused by sciatic nerve injury can significantly impact the quality of life of patients. The M1 phenotype of microglia has been reported to promote the progression of NP. Procaine is a lipid-soluble local anesthetic drug that exerts narcotic analgesic effects. Nevertheless, the detailed effect of procaine in NP is not clear. In order to explore the role of procaine in the polarization of NP microglia, HAPI cells were exposed to LPS to polarize into M1 type. In addition, the number of the M1 phenotype of HAPI cells was assessed using flow cytometry. The binding site between CCL5 and STAT3 was explored using the dual luciferase assay. Furthermore, in vivo experiments were applied for testing the impact of procaine on NP. LPS significantly inhibited HAPI cell viability, which was reversed by procaine. Consistently, procaine alleviated LPS-induced upregulation of inflammatory factors. Additionally, it significantly inhibited HAPI cell M1 polarization induced by LPS. Meanwhile, overexpression of STAT3 was able to promote HAPI cells M1 polarization through binding with the CCL5 promoter region and activating the PI3K/Akt signaling. Procaine could alleviate the painful behavior of complete Freund's adjuvant (CFA) rats by modulating the STAT3/CCL5 axis and inhibiting microglia M1 polarization. In conclusion, procaine alleviated the painful behavior of CFA rats via regulating the STAT3/CCL5 axis and inhibiting microglia M1 polarization. Hence, the research might provide a novel agent for NP treatment.

## Significance Statement

Neuropathic pain (NP) refers to the pain caused by the damage to the somatosensory system, which can be caused by brain or spinal cord injury and have a serious impact on the patient's quality of life. The M1 phenotype of microglia plays a crucial role in promoting the progression of NP. In this study, we investigated the specific mechanism of local anesthetic procaine in improving NP by inhibiting microglia polarization toward the M1 type. Our findings may provide a new drug for NP treatment.

## Introduction

Neuropathic pain (NP) refers to the pain induced by somatosensory system injury, and it is able to be caused by brain or spinal cord injury (Baron et al., 2010). NP is separated into central NP, such as multiple sclerosis, spinal cord injury, Parkinson's pain, poststroke pain, etc., and peripheral NP, which includes conditions like diabetic neuralgia and trigeminal neuralgia (Bouhassira, 2019). The clinical features in NP include allodynia and spontaneous pain, which can significantly impact patients’ mental health and overall quality of life and often lead to anxiety, sleep disorders, and depression (Gierthmuhlen and Baron, 2016). At present, the incidence of NP is ∼10%. With the increase in diabetes incidence and the age of the population, the incidence of NP will further increase (Bannister et al., 2020). Thus, to explore a novel therapeutic strategy for NP treatment is urgent.

Procaine is an anesthetic that can stabilize cell membranes. Hence, when the nerve impulse reaches, sodium and potassium ions are not able to move in and out of the cell membrane to generate depolarization and action potentials (Kurland and Hayman, 1976). However, procaine is not ideal for surface anesthesia due to its limited ability to penetrate the mucosa. Nonetheless, due to its advantages such as lower toxicity and precise efficacy compared with morphine, it is suitable for block anesthesia, osmotic anesthesia, and epidural anesthesia (Doud, 1951; Lesinski et al., 1973). More importantly, procaine can be hydrolyzed by esterase in plasma, leading to the formation of diethylaminoethanol and para-aminobenzoic acid. The presence of para-aminobenzoic acid may interfere with the impact of sulfonamides, and then caution should be exercised when using procaine in combination with sulfonamides (Fischbach et al., 1949; Goldberg and Kagan, 1950). Meanwhile, procaine exerted a narcotic analgesic function, which suggested that it had the potential to inhibit NP progression. Nevertheless, the detailed impact of procaine in NP is largely unknown.

Signal transducers and activators of transcription 3 (STAT3) is involved in multiple cancers and inflammation (Ibrahim et al., 2024; Xiyue and Jinyue, 2024). In addition, STAT3 is conformed to play a vital role in the polarization of microglia and neuroinflammation. For example, a previous report indicated that fisetin could promote functional recovery after spinal cord injury by inhibiting microglia/macrophage M1 polarization and the JAK2/STAT3 signaling pathway (Rong et al., 2024). In a rat model, the XIST/miR-544 axis promoted NP by activating STAT3 (Jin et al., 2018). It is worth noting that procaine attenuates the pain behavior of NP model rats by inhibiting JAK2/STAT3 signaling (Li et al., 2016). Thus, this study aimed to investigate the relationship between procaine and STAT3 in the polarization of microglia during the progression of NP. Cysteine-cysteine motif ligand 5 (CCL5) is a small-molecule protein expressed in the blood (X. F. Zhang et al., 2023a). Studies have shown that CCL5 is associated with NP, and blocking CCL receptor 5 could inhibit paclitaxel-induced peripheral NP caused by increased deoxycholic acid (Zhong et al., 2023). In addition, targeting CCL5 signaling has been shown to reduce neuroinflammation after seizures (Z. Zhang et al., 2023b). Moreover, CCL5 could induce a proinflammatory profile in microglia in vitro (Skuljec et al., 2011). However, whether CCL5 can regulate the pain behavior of complete Freund's adjuvant (CFA) rats by affecting microglia polarization has not been reported, which is worthy of further study. The research conducted by Chou et al. indicated that CCL5 could lead to hypothalamic insulin signaling for systemic insulin responsiveness through activation of PI3K/Akt signaling (Chou et al., 2016). Besides, PI3K/Akt signaling was widely distributed in spinal microglia (Hu et al., 2023). From the prediction of JASPAR (http://jaspar.genereg.net/), STAT3 had binding sites with the CCL5 promoter region; STAT3 may transcriptionally upregulate CCL5 and activate PI3K/Akt signaling to promote M1 polarization of microglia, thereby enhancing neuroinflammation.

Based on the above research, it could be hypothesized procaine might inhibit the progression of NP by regulating STAT3/CCL5 axis to inhibit M1 polarization of microglia. Hence, this work aimed to assess the role of procaine on NP, and the present research would bring a new therapeutic strategy for the treatment of NP.

## Material and Methods

### Cell culture

Microglia (HAPI) originated from ATCC and were plated in DMEM medium (Life Technologies) containing FBS (10%, Invitrogen). Cells were maintained in the condition of 5% CO_2_ at 37°C. To simulate NP in an in vitro model, the HAPI cells were added with 1.25, 2.5, 5, 10, and 20 ng/ml LPS for 24 h. For investigating the impacts of procaine on NP, cells were exposed to 2, 8, and 16 μg/ml procaine for 24 h. Procaine originated from Sigma-Aldrich.

### Cell transfection

In order to obtain STAT3 overexpressing cells, pcDNA3.1-STAT3 or pcDNA3.1 plasmids obtained from GenePharma were employed for transfecting HAPI cells with Lipofectamine 2000 (Invitrogen). The transfections were carried out for 48 h. Cells were transfected with CCL5 shRNA1 (sh-CCL5-1, GenePharma), CCL5 shRNA2 (sh-CCL5-2, GenePharma), CCL5 shRNA3 (sh-CCL5-3, GenePharma), STAT3 shRNA1 (sh-STAT3-1, GenePharma), STAT3 shRNA2 (sh-STAT3-2, GenePharma), and STAT3 shRNA3 (sh-STAT3-3, GenePharma) by Lipofectamine 2000 for 48 h; STAT3 or CCL5 knockdown cells were established.

### Western blot

RIPA (Beyotime) was applied for isolating protein from tissues or cells. BCA kit was employed in protein quantification. SDS–PAGE (10%) was employed for separating protein (40 μg/lane). Subsequently, separated proteins were transferred onto PVDF membranes. Primary antibodies were applied for incubating the membranes overnight after blocking for 1 h with 5% skimmed milk as follows: anti-STAT3 (R&D Systems; 8905-GT-020, 1:1,000), anti-CCL5 (Santa Cruz Biotechnology; sc-393006, 1:1,000), anti-STAT6 (Abcam; ab32108, 1:1,000), anti-IL-4 (Abcam; ab62351, 1:1,000), anti-PI3K (Abcam; ab46154, 1:2,000), anti-p-PI3K [Cell Signaling Technology (CST); 12938, 1:2,000], anti-p-Akt (CST; 12938, 1:2,000), anti-Akt (CST; 12938, 1:2,000), anti-STAT1 (Abcam; ab234400, 1:1,000), anti-Nav1.2 (Abcam; ab132328, 1:1,000), Nav1.3 (Abcam; ab66743, 1:1,000), Nav1.6 (Abcam; ab181759, 1:1,000), and anti-β-actin (Abcam; ab8226, 1:1,000). Afterward, secondary antibodies (HRP-conjugated, Abcam; ab288151, 1:5,000) were used for incubating the membranes for 1 h. ECL kit was used for visualizing protein bands. Exposure was performed using a gel imaging system. The results were calculated using the ImageJ software.

### RT-qPCR

TRIzol (Takara Bio) was applied for extracting RNA from tissues or cells. PrimeScript Kit (Takara Bio) was employed for synthesizing cDNA. SYBR methods were used in RT-qPCR with the ABI7500 system. RT-qPCR was employed as following described: 94°C for 2 min, followed by 35 cycles (94°C for 30 s and 55°C for 45 s). The primers originated from GenePharma. The 2^−ΔΔCT^ method was applied in data quantification. The primers originated from GenePharma: STAT3, F, 5′-CTCATCCGACTTGCAAGTCCCT-3′ and R, 5′-CTCGTCCGGCCACCTTGTCTCCAA-3′; β-actin, F, 5′-GTCCACCGCAAATGCTTCTA-3′ and R, 5′-TGCTGTCACCTTCACCGTTC-3′; and CCL5, F, 5′-GGACTATGTATTGGTCCCTACCG-3′ and R, 5′-TCGATGGTTGCAATGGTGTC-3′.

### ELISA

TNF-α, IL-β, and IL-6 levels in cell supernatants or tissues were assessed using ELISA kits (MedChemExpress). The corresponding absorbance was read on the SuPerMax 3000AL microplate reader (Shanpu Biotech) at 450 nm.

### MTT assay

HAPI cells were plated at a density of 5 × 10^3^ cells/ml in a culture plate (96-well) overnight. After treatment for 24 h, cells were exposed to 20 μl MTT for another 4 h. The supernatants were then removed, and the plates were added with DMSO (200 μl). The absorbance (490 nm) was assessed with a microplate reader.

### Flow cytometry

The distribution of CD86 and CD206 was detected using flow cytometry to determine the ratio of M1/M2 microglia. SYTOX Blue dead cell staining (Invitrogen) was performed to gate out dead cells. Cells (1 × 10^5^) were suspended, and then the single-cell suspensions were incubated, at 4°C for 30 min. Next, cells were stained with anti-CD86, anti-CD206, and anti-IBα1 APC (BioLegend). After that, CytoFix/Cytoperm kit (BD Pharmingen) was applied for permeabilizing cells after cells were fixed, and then a FITC-conjugated secondary antibody was used to stain cells. The cells were gated on IBα1- and CD86-positive expression, which were identified as the M1 microglia. In addition, the cells were gated on IBα1- and CD206-positive expression, which were identified as the M2 microglia. Unstained and fluorescein-conjugated isotypic cells served as the controls. The cells were washed twice with cell staining and then sorted and assessed by FACS, using a MoFlo XDP High-Performance Cell Sorter (Beckman Coulter). The data were acquired and analyzed using the IDEAS software (EMD Millipore), with representative images shown in the figure.

### Dual luciferase reporter assay

CCL5 WT was constructed by inserting the CCL5 sequence containing the STAT3 binding site into the pGL3-basic luciferase reporter vector, and CCL5 MUT was generated by mutating the predicted site. The CCL5 WT sequence is “attttgggaagc,” while the corresponding CCL5 MUT sequence is “cggggtttcctc.” The above sequence originated from GenePharma. The WT or MUT CCL5 vectors were applied to transfect cells with NC or STAT3 overexpression vector with Lipofectamine 2000 (Invitrogen) for 48 h. Dual-Glo Luciferase system was applied for detecting luciferase activities.

### In vivo experiments

Beijing Vital River provided the Wistar rats (male, 120–150 g). Animals were kept for 2 weeks to rule out the infections before experiments. Rats were housed in conditions of 55.5%, RT of 22°C, and a 12 h light/dark cycle. The experiment was applied at the First Affiliated Hospital of Jiamusi University following the principles and guidelines of the NIH and Ethics Committee of the First Affiliated Hospital of Jiamusi University. Rats were classified into the following groups (Control, Model, Model + proca, Model + CSF1Ri, Model + CSF1Ri + proca). To mimic NP in vivo, 0.1 ml CFA (Sigma-Aldrich) was injected into rats under anesthesia (plantar surface in the left hindpaw) using isoflurane treatment (1.5–2.5%). Rats in the sham group were treated with an equal volume of saline in the same place. Subsequently, behavioral tests containing thermal sensitivity and mechanical sensitivity measurements were applied once every day to evaluate the model. Rats were administrated with procaine and/or CSF1R pharmacological inhibitor (CSF1Ri) intrathecally after CFA administration for 3 consecutive days. The rat tail-flick reflex suggested that the injected substance entered the subarachnoid space. Rats were killed using 3% pentobarbital sodium. Meanwhile, measurements of thermal withdrawal latency (TWL) and mechanical withdrawal threshold (MWT) were employed for evaluating the sensitivity of rats to pain. Rat tissues were collected for further analysis.

### Immunofluorescence staining

Triton X-100 (0.3%) was applied for permeabilizing the samples for 15 min after fixing the samples with 4% paraformaldehyde. Anti-CD86 (Abcam, 1:500), anti-IBα1 (Abcam, 1:50), and anti-CD206 (4 µl for 1 × 10^6^ cells) were applied for incubating the samples for 1 h. Subsequently, samples were incubated using secondary antibody (HRP-conjugated, Abcam, 1:5,000) for 1 h. The results were observed under a fluorescence microscope.

### Statistical analysis

Each group employed three replicated experiments and mean ± standard deviation (SD) was used in expressing data. One-way analysis of variance followed by Tukey's test (more than two groups, GraphPad Prism7) or Student's *t* test (only two groups) was employed for analyzing the differences. *p* < 0.05 indicates an obvious difference.

## Results

### LPS inhibited HAPI viability and upregulated inflammatory factors, STAT3 and CCL5 expression

**Figure 1.**
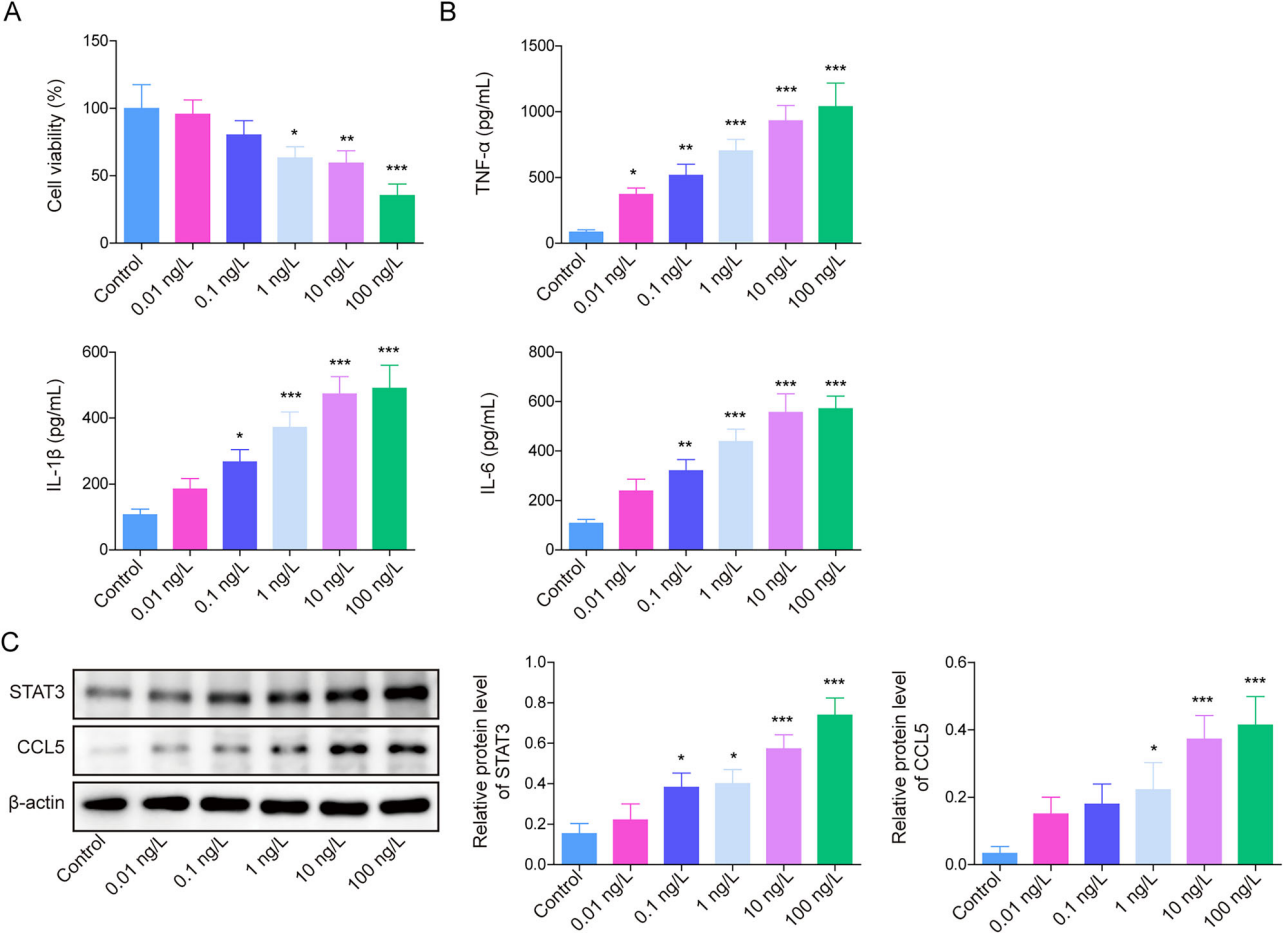
LPS inhibited HAPI viability and upregulated inflammatory factors, STAT3 and CCL5 levels. ***A***, The viability of HAPI cells was assessed using an MTT assay. ***B***, ELISA was applied to check TNF-α, IL-1β, and IL-6 levels. ***C***, The levels of STAT3 and CCL5 were assessed by a Western blot. Mean ± SD was used for expressing data. All data were obtained from at least three replicate experiments. *p < 0.05; **p < 0.01; ***p < 0.001.

To conduct this research, we first established an NP in vitro model; HAPI cells were added with various concentrations of LPS. As indicated in Figure 1*A*, it could be observed that the viability of HAPI cells decreased progressively with increasing doses of LPS. Furthermore, LPS treatment resulted in a dose-dependent increase of TNF-α, IL-1β, and IL-6 levels in the supernatants of HAPI cells (Fig. 1*B*). STAT3 and CCL5 levels in HAPI cells were found to increase upon LPS treatment (Fig. 1*C*). We observed that HAPI cell treatment with 10 ng/ml LPS resulted in a significant upregulation of STAT3 and CCL5 expression. Therefore, LPS of this concentration was applied for further experiments. In summary, LPS increased the inflammatory factors, STAT3 and CCL5 expressions in HAPI cells, while the cell activity of HAPI was inhibited.

### Procaine inhibited LPS-induced M1 polarization of HAPI cells by inhibiting STAT3

**Figure 2.**
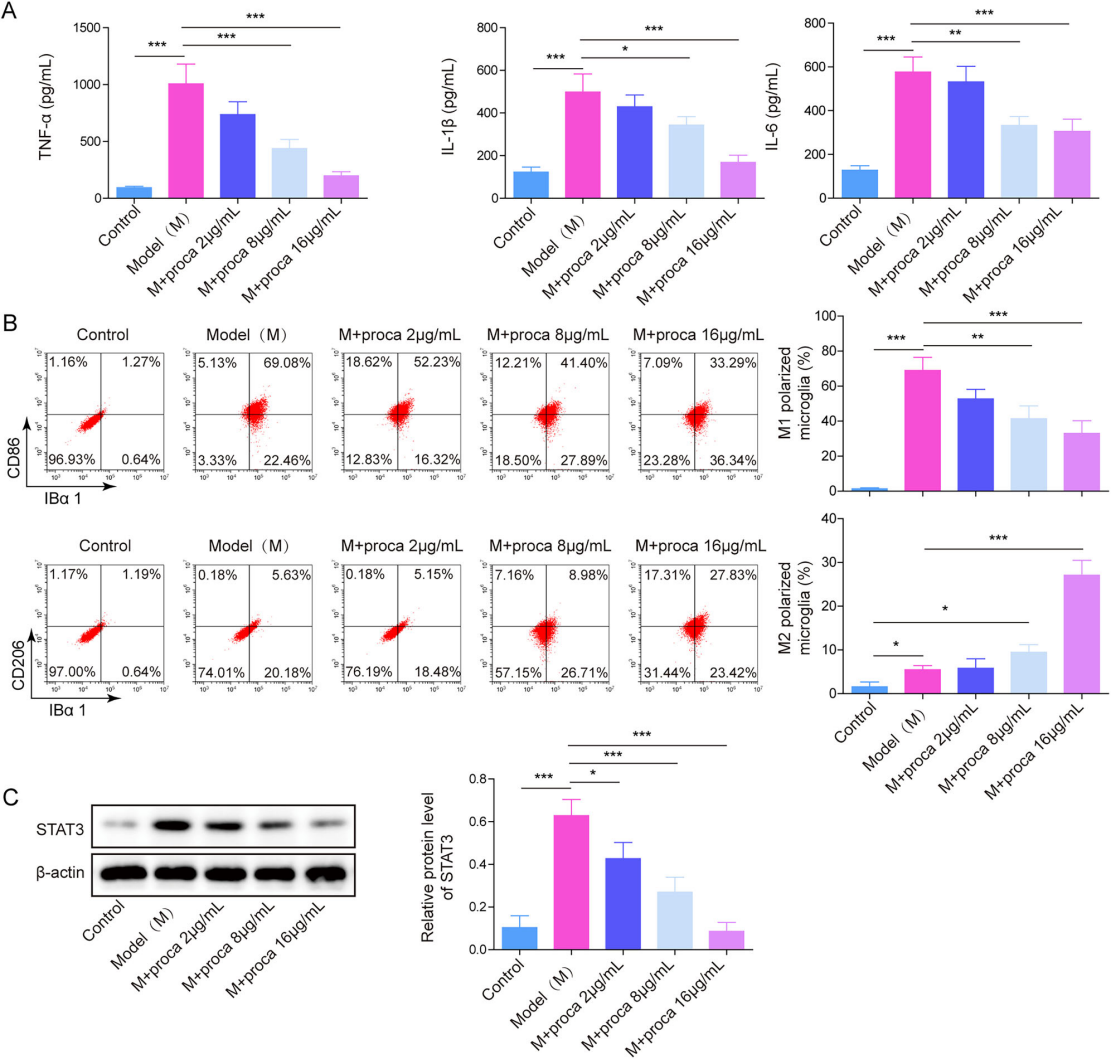
Procaine attenuated LPS-induced M1 polarization of HAPI cells by inhibiting STAT3. ***A***, TNF-α, IL-1β, and IL-6 levels were investigated using ELISA. ***B***, The distribution of M1/M2 phenotype microglia was tested by using flow cytometry. ***C***, A Western blot was employed for testing STAT3. The measurement data were presented as mean ± SD. All data were obtained from at least three replicate experiments. *p < 0.05; **p < 0.01; ***p < 0.001.

**Figure 3.**
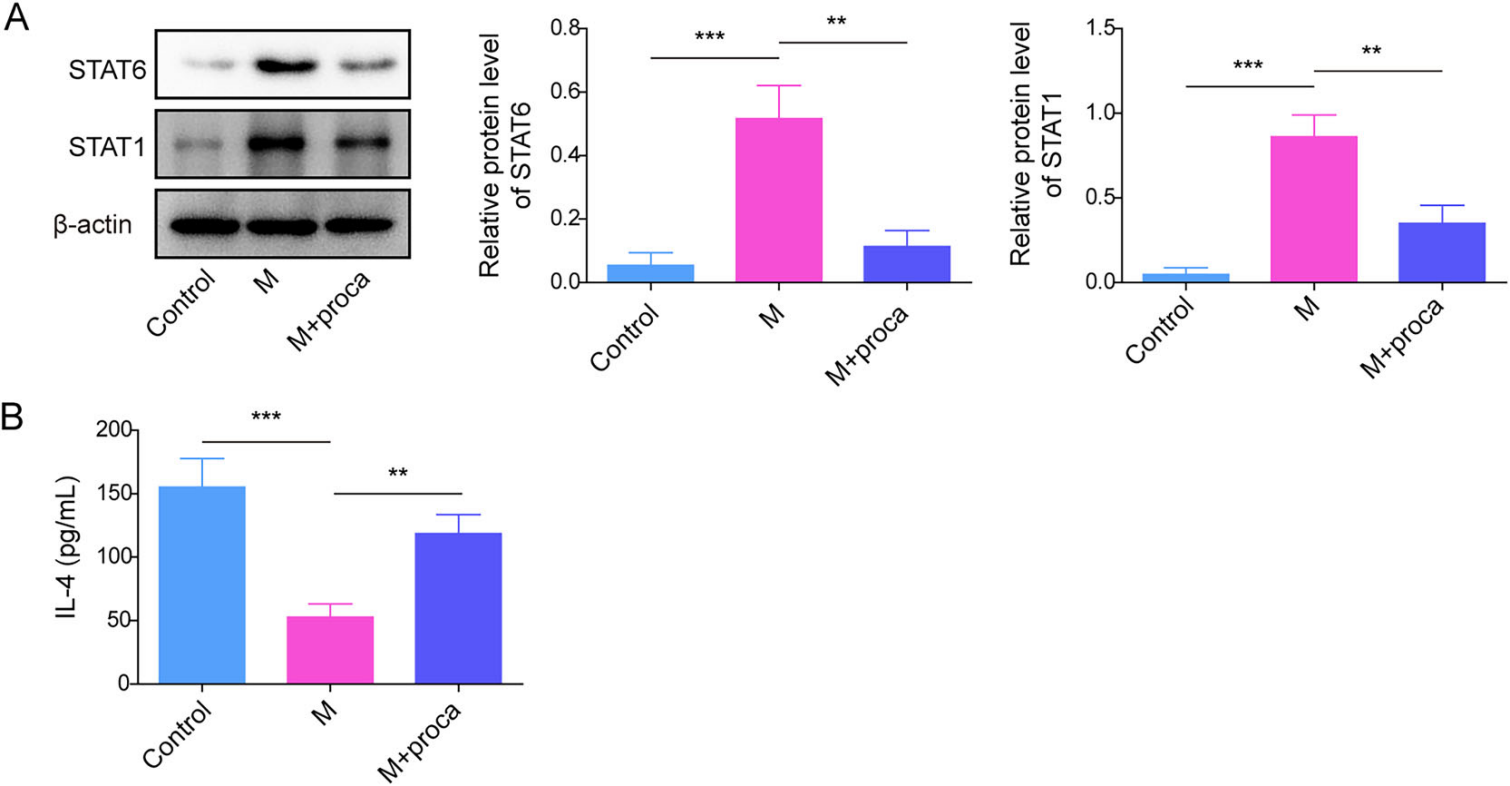
***A***, The STAT3 and STAT6 expressions in rats were tested using the Western blot. ***B***, The level of CCL4 was assessed by ELISA. ***C***, A Western blot was provided for measuring the level of CCL5 in HAPI cells transfected with CCL5 WT and CCL5 MUT. The measurement data were presented as mean ± SD. All data were obtained from at least three replicate experiments. **p < 0.01; ***p < 0.001.

**Figure 4.**
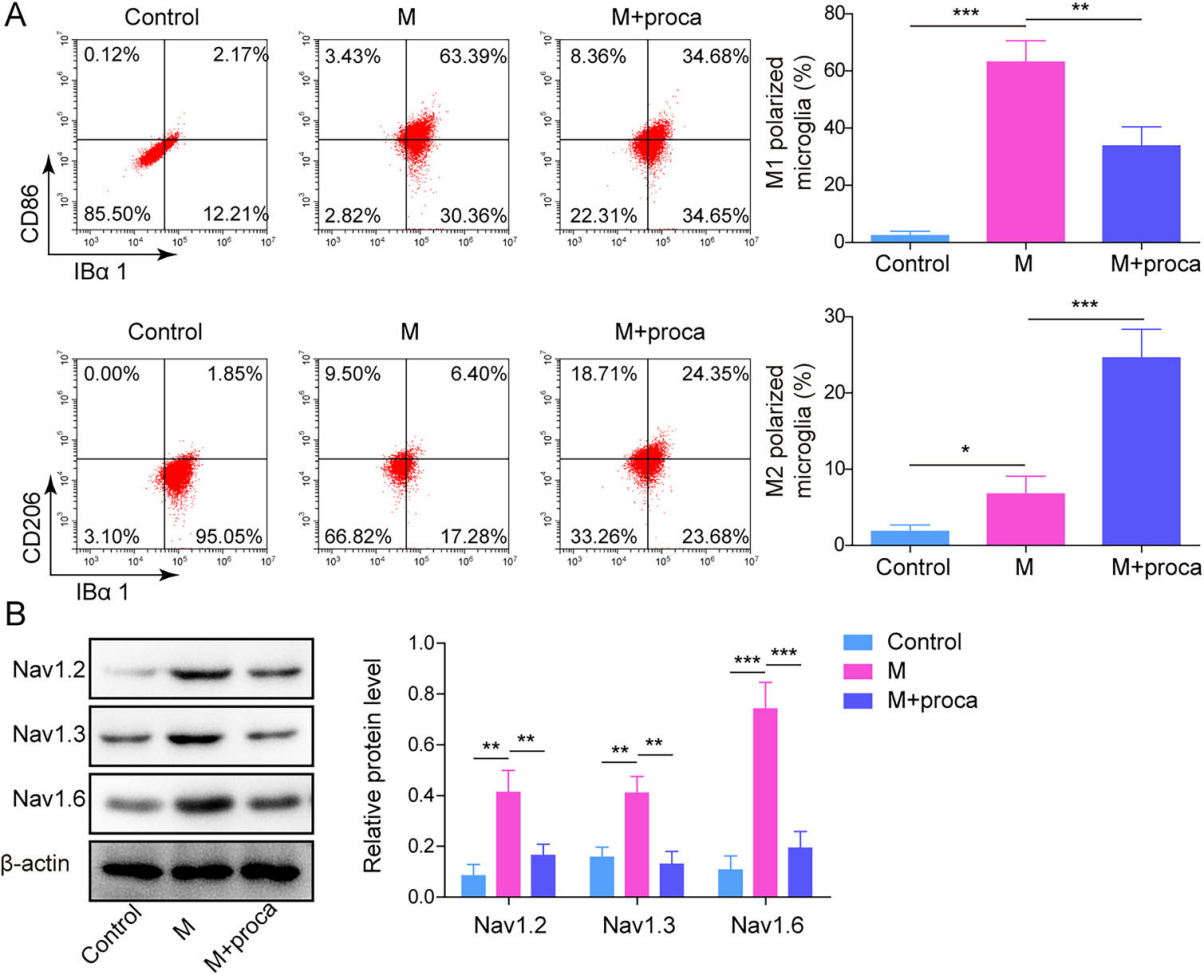
***A***, The distribution of M1/M2 phenotype microglia was tested by using flow cytometry. ***B***, The Western blot was used to detect Nav1.2, Nav1.3, and Nav1.6 levels in CFA rats. The measurement data were presented as mean ± SD. All data were obtained from at least three replicate experiments. *p < 0.05; **p < 0.01; ***p < 0.001.

To investigate the mechanism of procaine in NP, incubating LPS induced HAPI with different concentrations of procaine. In Figure 2*A*, TNF-α, IL-1β, and IL-6 levels were elevated in LPS-stimulated HAPI, but the promotion of LPS on inflammatory factors was alleviated after procaine administration. In addition, LPS significantly increased the ratio of M1-polarized microglia and decreased the ratio of M2-polarized microglia, while these phenomena were rescued by procaine (Fig. 2*B*). Consistent with the previous results, STAT3 was upregulated in LPS-induced cells, while it was significantly inhibited with an increase of the concentrations (4, 8, and 16 μg/ml) of procaine (Fig. 2*C*). According to the above data, 16 μg/ml procaine was selected in subsequent analysis. The STAT pathway-related factors STAT6, STAT1, and IL-4 were also detected, as shown in Figure 3, *A* and *B*, LPS obviously upregulated the levels of STAT6 and STAT1 and inhibited the expression of IL-4 in LPS-induced HAPI cells, while procaine partially alleviated the induction of STAT6 and STAT1 by LPS. In addition, considering the effect of procaine on voltage-gated channels, the connection between procaine and voltage-gated sodium channels in inflammatory response was investigated. Our results showed that the levels of sodium channel-related proteins (Nav1.2, Nav1.3, and Nav1.6) were significantly upregulated in LPS-induced HAPI cells and the M1 polarization HAPI cells were promoted. However, procaine obviously reversed this phenomenon (Fig. 4*A*,*B*). Taken together, procaine significantly inhibited LPS-induced microglia M1 polarization mainly through the downregulation of STAT3.

### Knocking down STAT3 mitigated LPS-induced HAPI M1 polarization by inhibiting PI3K/Akt signaling

**Figure 5.**
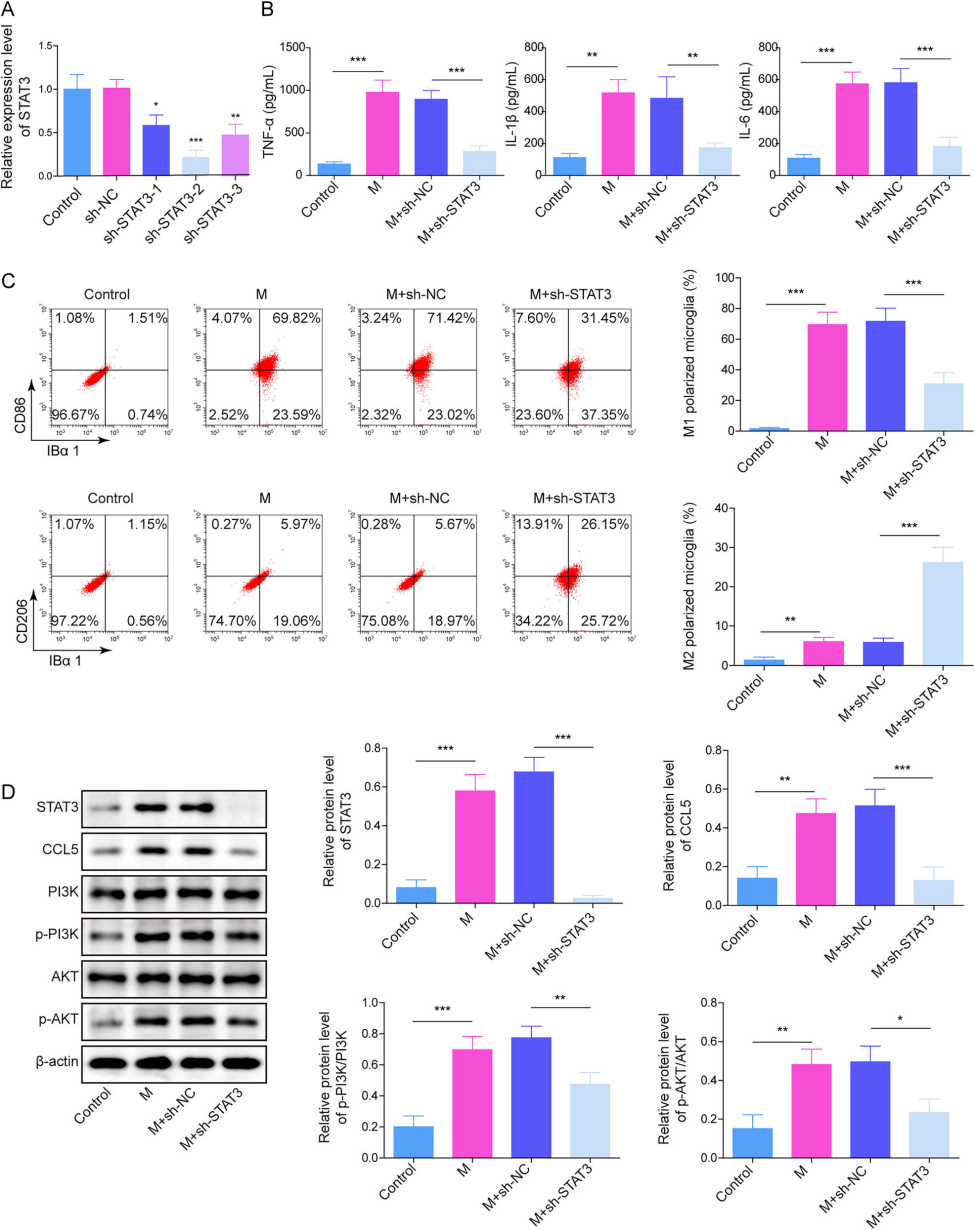
Knocking down STAT3 mitigated LPS-induced HAPI M1 polarization by inhibiting CCL5/PI3K/Akt. ***A***, The level of STAT3 was tested using RT-qPCR. ***B***, TNF-α, IL-6, and IL-1β levels were investigated using ELISA. ***C***, The distribution of M1/M2 phenotype microglia was tested with flow cytometry. ***D***, STAT3, CCL5, p-PI3K, PI3K, p-Akt, and Akt levels were assessed by a Western blot. The measurement data were presented as mean ± SD. All data were obtained from at least three replicate experiments. *p < 0.05; **p < 0.01; ***p < 0.001.

In order to test the function of STAT3 in M1 polarization of microglia, HAPI cells were exposed to STAT3 shRNA. The data showed the STAT3 level in HAPI cells was inhibited by STAT3 shRNAs (Fig. 5*A*). Since the better transfection efficiency of sh-STAT3-2 in HAPI cells than other shRNAs, sh-STAT3-2 was selected in the following experiments. LPS-caused upregulation of proinflammatory cytokines in HAPI cells significantly declined after STAT3 downregulation (Fig. 5*B*). In addition, LPS increased the population of M1 phenotype HAPI cells and decreased M2 phenotype HAPI cells, which were greatly reversed in the presence of STAT3 shRNA (Fig. 5*C*). Consistently, LPS notably upregulated the levels of STAT3 and CCL5 and promoted p-Akt and p-PI3K expressions in HAPI cells, while this phenomenon was obviously abolished by silencing STAT3 (Fig. 5*D*). To sum up, STAT3 inhibition reversed LPS-induced M1 polarization in microglia by blocking PI3K/Akt signaling.

### Procaine alleviated LPS-induced HAPI M1 polarization by inhibiting the STAT3/CCL5/PI3K/Akt axis

**Figure 6.**
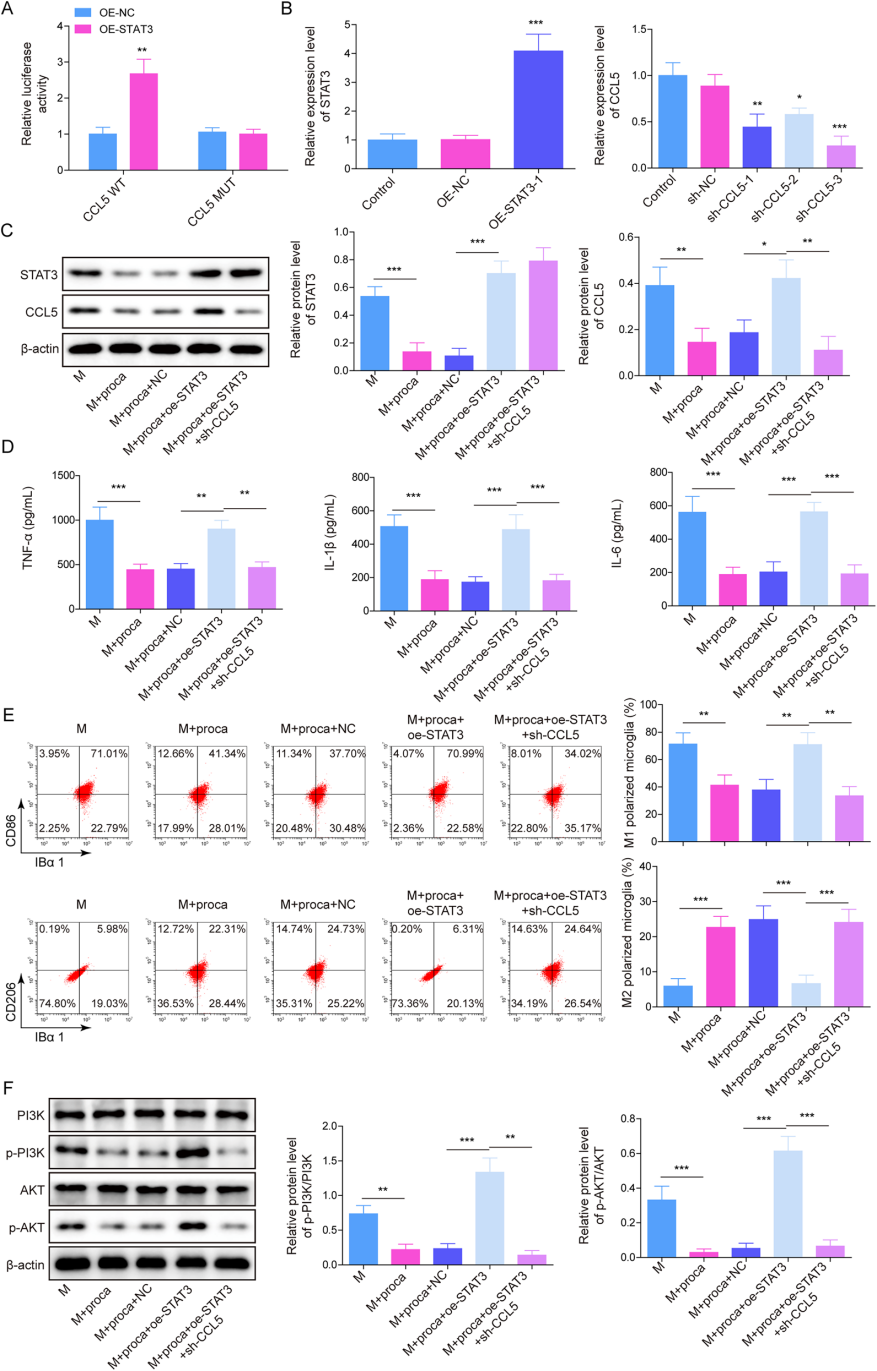
Procaine alleviated LPS-induced HAPI M1 polarization by inhibiting the STAT3/CCL5/PI3K/Akt axis. ***A***, The interaction between STAT3 and CCL5 was analyzed using a dual luciferase assay. ***B***, ***C***, The levels of STAT3 and CCL5 were examined using RT-qPCR and Western blot. ***D***, ELISA was applied to check TNF-α, IL-1β, and IL-6 levels. ***E***, Flow cytometry was provided for detecting the distribution of M1/M2 phenotype microglia. ***F***, p-Akt, Akt, p-PI3K, and PI3K levels were assessed using the Western blot. The measurement data were presented as mean ± SD. All data were obtained from at least three replicate experiments. *p < 0.05; **p < 0.01; ***p < 0.001.

From the prediction of JASPAR, there is a binding site between STAT3 and CCL5 promoter region. As shown in Figure 6*A*, STAT3 overexpression notably increased the luciferase activity in CCL5 WT, which indicated that STAT3 could transcriptionally activate CCL5. To further confirm the role of STAT3/CCL5 axis in procaine-mediated microglia M1 polarization, LPS induced HAPI cells to M1 polarization and then transfected with CCL5 shRNA and pcDNA3.1-STAT3, followed by incubation with procaine. The transfection efficiency was illustrated in Figure 6, *B* and *C*, LPS elevated STAT3 and CCL5 levels in HAPI cells, whereas procaine inhibited their expressions. However, the overexpression of STAT3 counteracted the suppressive effect of procaine on STAT3 and CCL5, and the CCL5 level was notably inhibited in the presence of CCL5 shRNA. Overexpression of STAT3 increased TNF-α, IL-1β, and IL-6 levels in procaine and LPS cotreated HAPI cells, which was suppressed by CCL5 knockdown (Fig. 6*D*). Similarly, STAT3 upregulation increased the proportion of M1 phenotype HAPI cells under LPS, whereas knocking down CCL5 could reverse this process. The proportion of M2 phenotype HAPI cells was opposite to M1 phenotype HAPI cells under the above conditions (Fig. 6*E*). In addition, the procaine's suppression on p-Akt and p-PI3K levels in LPS-stimulated HAPI cells was reversed by STAT3 upregulation, while CCL5 shRNA transfection notably rescued STAT3 overexpression-induced upregulation of p-Akt and p-PI3K (Fig. 6*F*). All the data demonstrated that procaine inhibited LPS-induced HAPI cell M1 polarization by mediation of STAT3/CCL5/PI3K/Akt axis.

### Procaine alleviated the painful behavior in CFA rats

**Figure 7.**
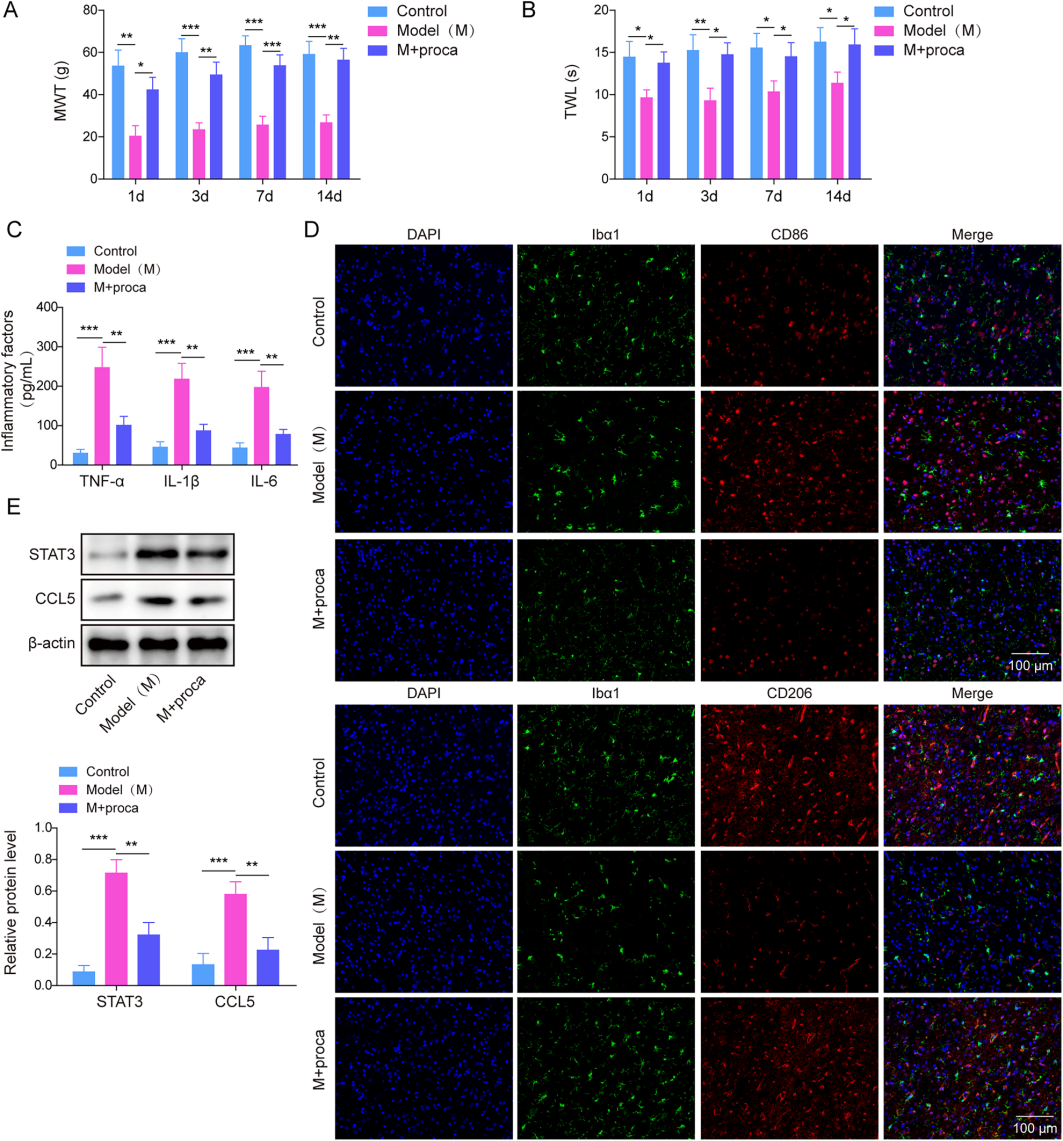
Procaine alleviated the painful behavior in CFA rats. ***A***, ***B***, MWT and TWL of rats were examined using behavioral testing. ***C***, The IL-6, TNF-α, and IL-1β expression in rats were tested using ELISA. ***D***, CD206 and CD86 levels in Wistar rat's spinal dorsal horn were examined by immunofluorescence staining. Scale bar, 100 μm. ***E***, Western blot was provided for measuring the levels of STAT3 and CCL5 in Wistar rat's spinal dorsal horn. The measurement data were presented as mean ± SD. All data were obtained from at least three replicate experiments. *p < 0.05; **p < 0.01; ***p < 0.001.

**Figure 8.**
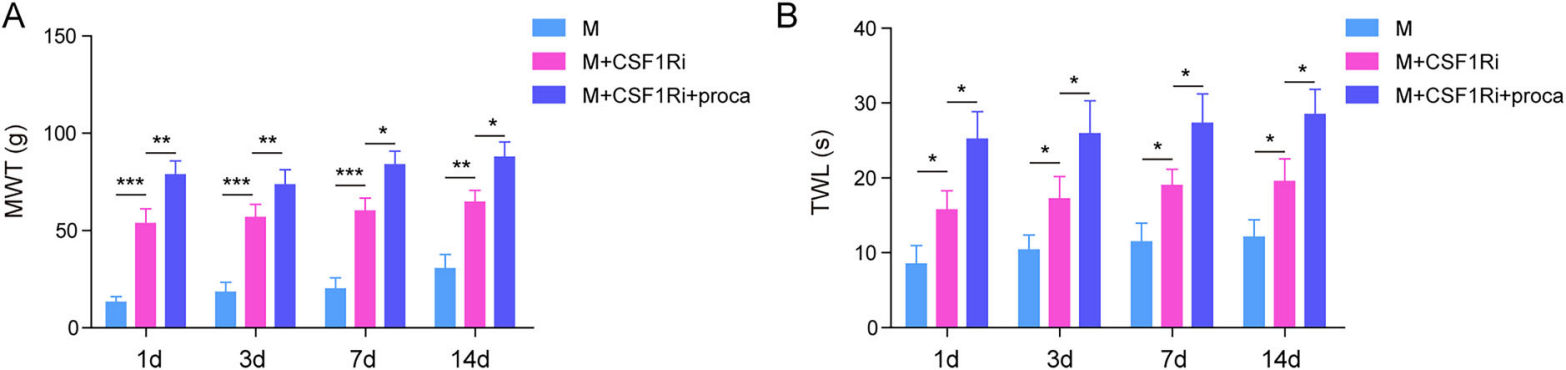
***A***, ***B***, MWT and TWL of rats were examined using behavioral testing in procaine and/or CSF1Ri-treated CFA rats. The measurement data were presented as mean ± SD. All data were obtained from at least three replicate experiments. *p < 0.05; **p < 0.01; ***p < 0.001.

Next, we verified the improvement of procaine on pain behavior in vivo; CFA was used to induce the NP model in Wistar rats. Subsequently, we conducted tests to measure the MWT and TWL on the 1st, 3rd, 7th, and 14th days following successful modeling. In Figure 7, *A* and *B*, the hindpaw MWT and TWL of rats were significantly suppressed by CFA, while after the administration of procaine, the MWT and TWL were partially restored in CFA-induced rat's hind paws. Consistent with the previous results, CFA treatment significantly upregulated inflammatory factors including TNF-α, IL-1β, and IL-6, whereas procaine alleviated the proinflammatory effect of CFA (Fig. 7*C*). CFA treatment elevated the level of CD86 and inhibited the CD206 level in Wistar rats spinal dorsal horn; it indicated an increase of M1 macrophages in CFA-treated rats. However, after administration with procaine, a reduction in the expression of CD86 was observed, indicating inhibition of M1 microglial polarization. On the contrary, CFA treatment reduced M2 macrophages in CFA-induced rats; however, after administration of procaine, an increase in polarization of M2 microglia was observed (Fig. 7*D*). Consistently, the expressions of STAT3 and CCL5 elevated in CFA-induced rat's spinal dorsal horn, while it was subsequently inhibited by procaine (Fig. 7*E*). On the other hand, CSF1Ri increased the MWT and TWL in CFA rats, which was further enhanced by procaine (Fig. 8*A*,*B*). Taken together, procaine alleviated the painful behavior of CFA rats through the suppression of the STAT3/CCL5 axis.

## Discussion

Previous research has shown that the M1 polarization of microglia is related to NP development. For instance, Zhang et al. found that DKK3 was able to alleviate NP progression by downregulating JNK/p-38–modulated M1 polarization (L. Q. Zhang et al., 2022); Wu et al. suggested that pharmacological inhibition of the cGAS-STING pathway could inhibit M1 polarization of microglia in the spinal cord and alleviate the development of NP (Wu et al., 2022). This study consistently demonstrated that procaine has the ability to relieve the painful behavior of rats by inhibiting the M1 polarization of microglia, Furthermore, our findings suggest that procaine can modulate the STAT3/CCL5 axis both in vivo and in vitro. Hence, we first confirmed the role of procaine in modulating STAT3 in the progression of NP. All the above outcomes suggested that procaine could attenuate the painful behavior of CFA rats by regulating the STAT3/CCL5 axis.

Procaine has been confirmed to be used as an anesthetic due to its ability to alleviate pain in cancer and inflammation patients (Kurland and Hayman, 1976; Chapman et al., 1992). In this research, it was discovered that procaine inhibited the STAT3/CCL5 axis, which in turn affected the PI3K/Akt signaling pathway. Interestingly, the PI3K/Akt pathway has been known to contribute to inflammatory pain induced by CFA injection; the inhibition of phosphorylated activation of the PI3K/Akt/mTOR pathway caused anti-inflammatory pain impact (Fan et al., 2021). Moreover, procaine has been shown to inhibit EMT by inhibiting the PI3K/Akt pathway (Yang et al., 2022). Meanwhile, Li et al. implied that procaine could inhibit the pain behaviors of NP rats possibly through downregulating JAK2/STAT3 signaling (Li et al., 2016). Our study shows that procaine plays a role in blocking the PI3K/Akt pathway, and it might be used for some inflammatory diseases other than analgesic anesthesia.

It was demonstrated that CCL5 was able to be a vital regulator in the progression of NP. Knockdown of CCL5 in mice was able to cause decreased local macrophage recruitment and behavioral hypersensitivity in an NP progression (Liou et al., 2012); Malon et al. suggested calcitonin gene-related peptide could lead to peripheral nerve injury-induced mechanical hypersensitivity through the CCL5 pathway (Malon and Cao, 2016). The prediction of JASPAR combined with the results showed that STAT3 could transcriptionally activate CCL5. Based on the above backgrounds, this finding suggests that STAT3 exacerbates the progression of NP by positively regulating CCL5. Additionally, this study has also revealed that knocking down CCL5 can reverse the upregulation of PI3K/Akt signaling and microglial M1 polarization induced by STAT3 overexpression. These findings collectively indicated that the STAT3/CCL5 axis may regulate microglia M1 polarization by mediating the PI3K/Akt pathway.

The voltage-gated sodium channels were confirmed to be widely distributed in microglia, and activation of voltage-gated sodium channels could lead to the excessive accumulation of Na in cells, thereby promoting the secretion of inflammatory factors and ultimately leading to intracellular inflammatory responses (Hossain et al., 2017). Local anesthetics are a class of heterogeneous compounds that block voltage-gated sodium channels (Nau and Wang, 2004). Our results showed that the levels of sodium channel-related proteins were significantly upregulated in LPS-induced HAPI cells and promoted M1 polarization HAPI cells. However, procaine obviously reversed this phenomenon; procaine might to some extent inhibit the polarization of microglia toward the M1 type by blocking voltage-gated sodium channels. Surprisingly, after removing microglia from CFA rats, it still plays an analgesic role after removing microglia in CFA rats. This may be due to the fact that immune cells are more activated and migrate to the nervous system than microglia during neuroinflammation. In addition, T cells and B cells play an important role in neuroinflammatory diseases by recognizing autoantigens or foreign antigens to activate immune responses (Schroeter et al., 2021). Therefore, procaine might alleviate CFA-induced pain response by regulating other immune cells. However, which immune cells are affected by procaine in CAF rats needs further study.

Indeed, some limitations in this research need to be addressed in future studies. Firstly, further validation is required to identify additional downstream targets of procaine apart from the STAT3/CCL5/PI3K/Akt axis. This will provide a more comprehensive understanding of procaine's pharmacology effect. Secondly, it is essential to confirm the relationship between STAT3/CCL5 and PI3K/Akt pathway, and more in-depth assessments are vital in the future.

In summary, procaine has been demonstrated to alleviate pain behavior in CFA rats via blocking the STAT3/CCL5 axis and inhibiting microglia M1 polarization. Thus, those findings suggest that procaine may have potential applications in clinical practice.

### Ethics approval and consent to participate

The experiment was applied at the First Affiliated Hospital of Jiamusi University following the principles and guidelines of the NIH and Ethics Committee of the First Affiliated Hospital of Jiamusi University.

### Availability of data and material

All data generated or analyzed during this study are included in this published article.

## Synthesis

Reviewing Editor: Jennifer Dulin, Texas A&M University Decisions are customarily a result of the Reviewing Editor and the peer reviewers coming together and discussing their recommendations until a consensus is reached. When revisions are invited, a fact-based synthesis statement explaining their decision and outlining what is needed to prepare a revision will be listed below. The following reviewer(s) agreed to reveal their identity: Zhongxiao Fu.

The authors have satisfactorily addressed all of the comments of the reviewers.

Reviewer's comments:

Reviewer #1:

The authors have addressed all of my questions in the revised manuscript thoroughly. I am supportive of its acceptance.

## Author Response

Your resubmission must include the manuscript with new text indicated in a bold or highlighted font. Separately, please provide a clean copy of the manuscript that includes the title page, which will be published if accepted. Please closely review your manuscript at this time for any final corrections in style or substance.

Dear editors and reviewers, Thank you for your letter and the constructive feedback provided by the reviewers regarding our manuscript titled "Procaine regulates the STAT3/CCL5 axis and inhibits microglia M1 polarization to alleviate CFA rats pain behavior". These comments were very helpful in revising and improving our paper. We have carefully considered these comments and have made corresponding corrections that we hope will be meet with your approval. These changes in the revised manuscript have been highlighted in yellow, a clean copy of the manuscript, including the title page, was also submitted simultaneously. Responses to the reviewers' comments have been provided below.

Synthesis of Reviews:

Computational Neuroscience Model Code Accessibility Comments for Author (Required):

N/A Synthesis Statement for Author (Required):

Synthesis statement:

The reviewers both agreed that microglia polarization is an important factor contributing to pain. However, several major questions were raised by the reviewers. There are concerns about how distinct M1/M2 types of microglia were identified, the mechanisms of procaine activity on microglia polarization, and the role of STAT3 and CCL5 in producing neuropathic pain. Please see below for a point-by-point critique.

Reviewers' comments:

Reviewer #1:

Microglia play an important role in initiating and maintenance of neuropathic pain through modulating inflammation and neuronal activity. Understanding the mechanisms by which microglia and neurons interact during neuropathic pain could constitute microglia as a new therapeutic target for the treatment of neuropathic pain. Anesthetic drugs, such as procaine and lidocaine, have been proven to relieve neuropathic pain (V Challapalli et al., 2005; IW Tremont-Lukats et al., 2005). One of the mechanisms for procaine impact on neuropathic pain is through JAK2/STAT3 signaling (D Li et al., 2016). In this paper, authors reported that procaine regulates CFA rats' pain through regulating STAT3/CCL5 axis and inhibiting microglia M1 polarization. This work is quite interesting study. However, there are some questions that need to be addressed.

Major points:

1, In Fig1 and Fig2, authors claim that LPS increased M1 microglia and decreased M2 microglia, which were rescued by procaine. How do authors identify M1 or M2 microglia? Response: Thanks very much for your kind comment. As revealed in Figure 1A -C, the levels of STAT3, CCL5 and inflammatory factors in HAPI cells were significantly upregulated by LPS. In addition, the ratio of CD86+Ibα1+ (M1 marker) and CD206+Ibα1+ (M2 marker) microglia was detected. As expected, the result indicated that LPS could increase M1 phenotype microglia while decreasing M2 phenotype microglia (Figure 2C). To make the conclusion more rational, we have supplemented images of M1 and M2 phenotypes microglia detected by flow cytometry in the figures. Please refer to the revised figures for details.

2, Procaine is well known to bind to and inhibit voltage gated sodium channels. How does procaine regulate M1/M2 microglia activation? Is it neuronal activation dependent? Does procaine have a direct impact on microglia activation? Response: Thanks very much for pointing out this. The voltage-gated sodium channels were confirmed to be widely distributed in microglia, and activation of voltage-gated sodium channels could lead to the excessive accumulation of Na in cells, thereby promoting the secretion of inflammatory factors and ultimately leading to intracellular inflammatory responses (PMID: 27655349). Local anesthetics are a class of heterogeneous compounds that block voltage-gated sodium channels. Therefore, we examined whether procaine can inhibit microglial inflammation by regulating voltage-gated sodium channels in the following experiments. Our results showed that the levels of sodium channel-related proteins (Nav1.2, Nav1.3 and Nav1.6) were significantly upregulated in LPS-induced HAPI cells, and the M1 polarization HAPI cells was promoted; However, procaine obviously reversed this phenomenon (Figure 7A-B). The above results indicated that procaine could to some extent inhibit the polarization of microglia towards the M1 type by blocking voltage-gated sodium channels.

3, CD68 is not a good marker to separate M1 versus M2 microglia. Authors should choose other markers to verify key findings related to M1/M2 microglia.

Response: Thanks very much for your valuable suggestion. According to what you mentioned above, we used CD86 as a typical biomarker for M1 microglia for further validation, and the number the number of CD86+Ibα1+ and CD206+Ibα1+ microglia was detected by flow cytometry. The results showed LPS increased M1 microglia and decreased M2 microglia.

4, How do STAT6 and IL4 levels change after LPS treatment with or without procaine? Response: Thanks very much for your question. As per your comment, the related experiments were performed. As shown in Figure 6A, LPS obviously upregulated the expression of STAT6, while procaine partially alleviated the induction of STAT6 by LPS. Moreover, LPS inhibited the level of IL-4 in HAPI cells, which was reversed by procaine (Figure 6B). Therefore, procaine also had a certain effect on the expressions of STAT6 and IL-4 in LPS-induced HAPI cells.

5, If authors deplete microglia in CFA rats pain model, dose procaine still have an impact on pain relief? Response: Thanks very much for pointing out this. According to what you mentioned above, CAF rats were administrated with CSF1R pharmacological inhibitor (CSF1Ri) to clear microglia. The MWT and TWL of CAF rats were detected on the 1st, 3rd, 7th and 14th days, respectively. The results showed that CSF1Ri increased the MWT and TWL in CFA rats, and procaine further enhanced the MWT and TWL in CSF1Ri-treated CAF rats (Figure 8A-B). The results suggest that procaine can still play an analgesic role after removing microglia in CFA rats. This may be because immune cells are more activated and migrate to the nervous system than microglia during neuroinflammation. In addition, T cells and B cells play an important role in neuroinflammatory diseases by recognizing autoantigens or foreign antigens to activate immune responses (PMID: 34691057). Therefore, procaine might alleviate CFA-induced pain response by regulating other immune cells. However, which immune cells are affected by procaine in CAF rats needs further study.

Reviewer #2:

Neuropathic pain is characterized by adverse events in neurons such like nerve fiber damage causing malfunctions (ectopic activity). This results in either peripheral or central sensitization depending on the primary disease condition. The observation for neuro-neuroinflammation, resulting in microglia polarization, had emerged as important factor contribution for this pathophysiology. In the manuscript evaluated, authors designed inflammatory microglia model in-vitro (lipopolysaccharide stimulation; LPS) and neuropathic pain model in-vivo (Complete Freund's adjuvant) to determine whether procaine inhibit neuropathic pain by modulating inflammatory microglia phenotype to ameliorate neuropathic pain. CCL5 and STAT3 were suggested, through open access transcription database, to inhibit neuro-inflammation polarization. Although the prepared manuscript argues for a rational generated by evidence and data driven hypothesis, the selected hypothesis and the outcomes are not strongly convincing.

Major concerns:

1. LPS is widely used in 100 ng - 1000 ng per milliliter range to stimulate microglia. Whether authors considered Zheng et al Cell Mol Neuro Biol 2012 using different TLR4 agonist that is similar to LPS induced microglial activation in HAPI cells with 200 ng/ml to investigate? What is the reason behind the dose difference? It can be clearly understood from the literature 100 ng -1000 ng/ml range is sufficient to induce inflammatory polarization in primary rat microglia. HAPI response presented in the manuscript at 10 microgram is relatively different. Taking into account from cell viability assay which supports cell survival, whether mg was a typing error for "ng"? Response: Thanks very much for your kind reminder. We apologize for our carelessness in labeling the units of LPS incorrectly in the manuscript and figures. In fact, we referred to previous research (PMID: 23642074) and designed a series of gradient concentrations (0, 1.25, 2.5, 5, 10, 20 ng/ml) to explore the effects of LPS on HAPI inflammation and cell viability. The results revealed that LPS of 10 ng/mL exhibited the most pro-inflammatory effect (Figure 1). Thus, 10 ng/mL of concentration was selected in subsequent analysis.

2. LPS-TLR4 induces STAT1 pathway for inflammation. What could be the fate of STAT1 in HAP1 LPS model.

Response: Thanks very much for pointing out this. Considering the role of the LPS-TLR4-induced STAT1 pathway on inflammation, we determined the effect of LPS induction on the expression of STAT1 in microglia in Figure S1A. The results showed that STAT1 was upregulated in LPS-induced microglia, but after the addition of procaine, the level of STAT1 was only partially downregulated. Therefore, in this study, procaine also has a regulatory effect on STAT1. However, whether it can suppress M1 microglia polarization and inflammatory response by inhibiting STAT1 in LPS-induced HAPI cells is unclear and needs further study.

3. How inflammatory (M1) and reversed inflammatory (M2) phenotypes were measured by flowcytometry is incomplete in methods section? How was percentage calculated? Whether the representation is only graph format? Isotype control over lapped images is missing. Whether live/dead cell were considered? Response: Thanks very much for your kind comment. The detailed contents of the methods in the measurement of M1/M2 macrophages were supplemented in the section of methods. The data were acquired and analyzed using IDEAS software (EMD Millipore, Seattle, WA), with representative images shown in the figure, and the percentage of M1 and M2 microglial cells can be seen in the images. In addition, SYTOX Blue dead cell staining (Invitrogen) was performed to gate out dead cells. Please refer to the revised manuscript for details.

3. How STAT3 and CCL5 are important for neuropathic pain? Current knowledge on STAT3 and CCL5 is not sufficiently detailed. What could be the fate of classically believed targets STAT1 or others, downstream LPS stimulation in the selected in-vivo model or cell line? How STAT3 and CCL5 are indispensable for neuropathic pain? Microglia activation in-vivo in the rat pain model does not increase Iba1 or CD68 staining to demonstrate neuro-inflammation and monocyte macrophage recruitment. How could authors suggest an increased TNF-a, IL-1b, IL-6 in rat model? Response: Thanks very much for your question. In our manuscript, we added evidence that STAT3 and CCL5 are involved in neuropathic pain. Please refer to the revised manuscript for more details. In addition, we further explored the effect of procaine on STAT1 in LPS-induced microglia, relevant data showed that LPS promoted the expression of STAT1 in HAPI cells, while procaine had a limited effect on it (Figure 6A). We chose the more representative M1 marker CD86 to re-test and improve the image quality of Figure 5D. The results showed that the level of CD86 was upregulated and the level of CD206 was downregulated in CFA rats, which proved that microglia in CFA rats were mainly pro-inflammatory phenotype M1 type. Correspondingly, increased inflammatory factors were detected in CFA rats.

5. How are WT and CCL5 mutant different in terms of genomic sequence? How were the cells generated? Are these cells characterized for bassline changes? Rational behind presenting mutant CCL5 could not conclude CCL5 played a major role.

Response: Thanks very much for pointing out this.CCL5 WT was constructed by inserting the CCL5 sequence containing the STAT3 binding site into the pGL3-basic luciferase reporter vector, and CCL5 MUT was generated by mutating the predicted site. The CCL5 WT sequence is "attttgggaagc", while the corresponding CCL5 MUT sequence is "cggggtttcctc". The WT or MUT CCL5 vectors were applied to transfect cells with NC or STAT3 overexpression vector with Lipofectamine® 2000 (Invitrogen) for 48 h. The related contents have been supplemented in the section of methods. Based on the above results, it could be inferred that STAT3 binds to the CCL5 promoter region and activates the transcription of CCL5, playing an important role in neuroinflammation. The promoter sequence was constructed before the firefly luciferase gene, and the transcription factor was overexpressed. When the transcription factor binds to the specific binding site on the promoter, the luciferase gene transcription is activated, so that the firefly luciferase can be expressed, and the final fluorescence intensity increases. When the binding site was mutated, the transcription factor could not bind to the promoter, and the fluorescence value did not change significantly. Therefore, the dual luciferase report assay can be used to detect the binding of STAT3 to the CCL5 promoter. Besides, WT or MUT CCL5 were not transfected into the cell genome, so there is no need to investigate their effects on CCL5 expression in HAPI cells.

6. To conclude STAT3/CCL5 axis is key for transitions to M1 and M2 is not convincing.

Response: Thank you very much for your question. It has been reported that HMGB1-induced CCL5 release from astrocytes was involved in promoting microglia/macrophage accumulation and M1 polarization (PMID：39333662). Moreover, CCL5 could induce a pro-inflammatory profile in microglia in vitro (PMID:21620385). In this study, we first found that procaine inhibited LPS-induced microglial polarization to M1 phenotype and blocked HAPI cell inflammation, which was reversed by STAT3 overexpression. Further experiments have shown that STAT3 transcriptionally activated CCL5, and CCL5 knockdown counteracted the promotion of STAT3 upregulation on inflammation and M1 phenotype polarization of HAPI cells in LPS and procaine co-treated HAPI cells. Therefore, we concluded that procaine could inhibit M1 microglia polarization by regulating the STAT3/CCL5 axis to alleviate neuroinflammation, thereby relieving NP in CFA rats.
